# Organic Vapor Sensing Mechanisms by Large-Area Graphene Back-Gated Field-Effect Transistors under UV Irradiation

**DOI:** 10.1021/acssensors.2c01511

**Published:** 2022-09-19

**Authors:** Katarzyna Drozdowska, Adil Rehman, Pavlo Sai, Bartłomiej Stonio, Aleksandra Krajewska, Maksym Dub, Jacek Kacperski, Grzegorz Cywiński, Maciej Haras, Sergey Rumyantsev, Lars Österlund, Janusz Smulko, Andrzej Kwiatkowski

**Affiliations:** † Department of Metrology and Optoelectronics, Faculty of Electronics, Telecommunications, and Informatics, Gdańsk University of Technology, G. Narutowicza 11/12, 80-233 Gdańsk, Poland; ‡ CENTERA Laboratories, Institute of High Pressure Physics PAS, 01-142 Warsaw, Poland; § Centre for Advanced Materials and Technologies CEZAMAT, Warsaw University of Technology, 02-822 Warsaw, Poland; ∥ Institute of High Pressure Physics PAS, Sokołowska 29/37, 01-142 Warsaw, Poland; ⊥ Department of Materials Science and Engineering, The Ångström Laboratory, Uppsala University, P. O. Box 35, 75103 Uppsala, Sweden

**Keywords:** graphene sensor, organic vapors, acetonitrile, tetrahydrofuran, chloroform, fluctuation-enhanced sensing, UV irradiation

## Abstract

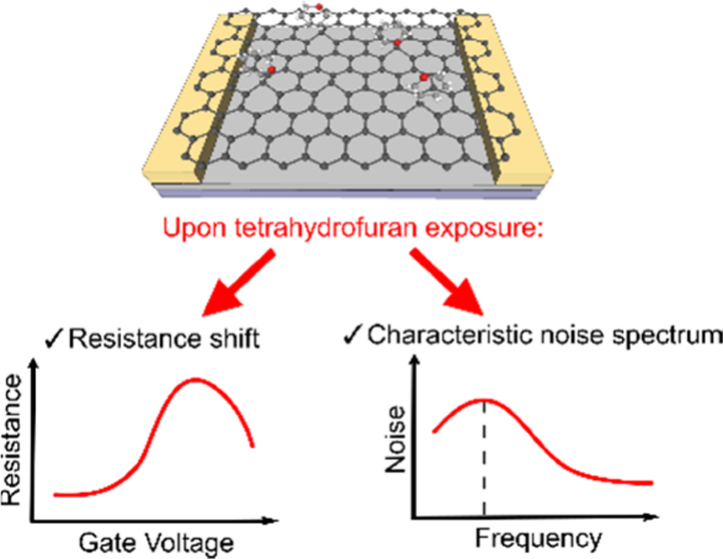

The gas sensing properties of graphene back-gated field-effect transistor (GFET) sensors toward acetonitrile, tetrahydrofuran, and chloroform vapors were investigated with the focus on unfolding possible gas detection mechanisms. The FET configuration of the sensor device enabled gate voltage tuning for enhanced measurements of changes in DC electrical characteristics. Electrical measurements were combined with a fluctuation-enhanced sensing methodology and intermittent UV irradiation. Distinctly different features in 1/*f* noise spectra for the organic gases measured under UV irradiation and in the dark were observed. The most intense response observed for tetrahydrofuran prompted the decomposition of the DC characteristic, revealing the photoconductive and photogating effect occurring in the graphene channel with the dominance of the latter. Our observations shed light on understanding surface processes at the interface between graphene and volatile organic compounds for graphene-based sensors in ambient conditions that yield enhanced sensitivity and selectivity.

## Introduction

Two-dimensional (2D) materials, notably graphene, have gained much attention due to their unique physical properties, special surface properties, high surface-to-volume ratio, unique charge transfer properties, and sensitivity to ambiance such as environmental conditions or electromagnetic irradiation.^1^ There has been significant progress in 2D material-based sensing devices over the last decade, including fabrication routes, doping or manufacturing of hybrid structures, and ways to enhance the signal response by means of, *e.g*., temperature or illumination enhancement.^2−4^ Among 2D materials, graphene and its derivatives are considered one of the most promising candidates for ultrasensitive gas sensors. Moreover, defective graphene monolayers exhibit more binding sites for target gas molecules, so simplified fabrication methods can be even more favorable for obtaining nonideal layers for highly sensitive devices.^5,6^

The nature of graphene makes its surface extremely active since all of the carbon atoms in the monolayer are exposed to the surrounding atmosphere. Molecular adsorption events caused by volatile compounds lead to local changes in the electrical properties, *e.g*., electrical conductance. Thus, graphene devices may be used as sensors based on a resistor or field-effect transistor (FET) configuration. Additional gate voltage modulation of the graphene in the FET configuration may enhance the sensitivity when DC responses are more pronounced in the specific gate voltage ranges.^7^ Several strategies for accelerating surface processes on graphene and related materials have been reported. Faster response and recovery times and more pronounced changes in sensor response have been obtained by employing elevated temperatures, dopants, cocatalysts, or irradiation in the ultraviolet (UV) spectral range.^8−10^ The energy provided by heat or UV LED irradiation can trigger the adsorption of molecules from the gas phase or desorption of previously attached species during the recovery time. UV irradiation at specific wavelengths may result in partial surface cleaning or generating weakly bonded oxygen ions, preparing the active surface for further gas detection.^7^ However, the complexity of the physicochemical processes behind the interactions at the interface when several external parameters are employed requires further investigation before using graphene-based materials as reliable sensors.

Unfortunately, employing only DC resistance measurements results in the low selectivity of pure graphene sensors because similar responses can be observed for different gases. One of the ways to achieve higher selectivity is to employ low-frequency noise measurements. Fluctuation-enhanced sensing (FES) utilizes information about the power spectral density of resistance fluctuations at low frequencies where 1/*f* noise (flicker noise) dominates.^11−14^ Gas molecules adsorbed on the surface of graphene or other low-dimensional materials create scattering and trapping centers. This leads to fluctuations in the number of charge carriers and their mobility.^15−17^ If a specific molecule creates just one type of center, it is characterized by a single characteristic time constant and Lorentzian shape of the noise spectrum. In this case, exposure to different gases results in different characteristic frequencies of the Lorentzian type of spectra. This gives rise to the higher selectivity of graphene sensors.^14,18^ Overall, along with DC electrical measurements, the FES method may enhance graphene-based sensor performance and provide additional information about the detected gases.

While results on detection of volatile organic compounds (VOCs) by small-area graphene monolayers have shown the potential to distinguish between various organic gases with graphene-FET devices,^19,20^ little research has been conducted to show the influence of sensor surface fabrication methods, pre-processing at high temperatures, and UV irradiation. It is known that the fabrication process determines the types of imperfections on the graphene surface, *e.g*., oxygen and carboxylic groups, which may be crucial for the adsorption and desorption of molecules. Therefore, the observed resistance fluctuations under environmental exposure could be related to the way of graphene fabrication. An imperfect yet well-characterized graphene layer can – apart from being more sensitive and selective – simplify sample preparation requirements and reduce fabrication costs. Therefore an important question is how technological graphene preparation affects the sensor response.

Herein, we present studies of graphene-based FET (GFET) sensors for the detection of selected VOCs, focusing on explaining possible detection mechanisms. We combine DC resistance measurements (sensor resistance between the drain and source as a function of gate voltage) and flicker noise measurements collected at room temperature (RT) in the presence of selected gases for UV irradiated and nonirradiated sensors. We study GFET responses toward selected volatile organic gases after cleaning graphene at high temperatures and in a vacuum. We show that the cleaning procedure allows graphene sensor preparation that yields more sensitive gas detection by reducing the influence of humidity and pollutant molecules adsorbed on the surface from laboratory air. Finally, we present the results of graphene aging and the possibility of refreshing the sensors by employing UV irradiation and inert gas purging.

## Results and Discussion

### GFET Sensor Characterization

**Figure 1:**
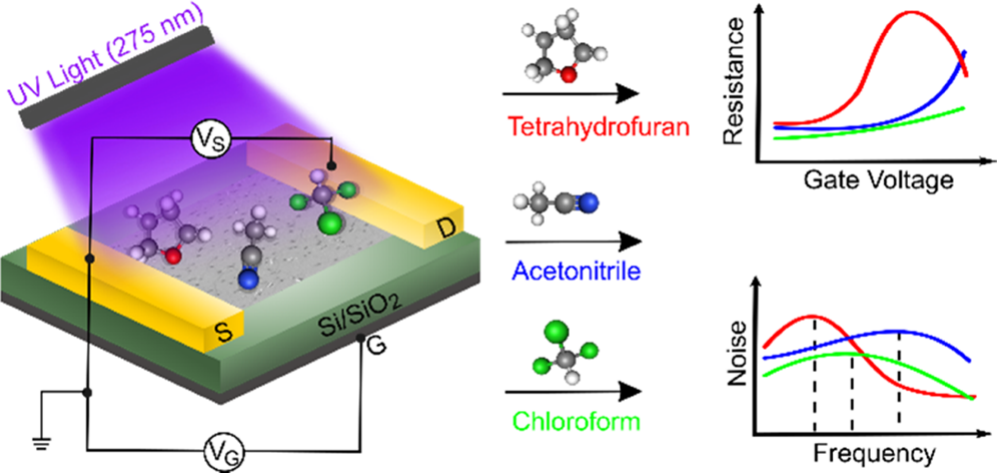
Schematic representation of the investigated GFET (left panel) structure with the proposed sensing analysis (right panels).

DC resistance vs gate voltage and fluctuation-enhanced sensing were employed to study the gas-induced response of the GFETs to tetrahydrofuran, acetonitrile, and chloroform, as schematically shown in Figure 1. The presence of tetrahydrofuran, acetonitrile, and chloroform molecules in the sensor′s ambiance produces characteristic changes in the resistance vs gate voltage characteristics and noise spectra, as depicted illustratively on the right side of Figure 1. The sensor response to acetone vapor was also investigated to compare it with results for tetrahydrofuran, as a molecule comprises the same elements but at different structural configurations. Tetrahydrofuran, acetonitrile, and chloroform are widely used organic solvents that vary in their chemical properties (*e.g*., chemical composition, electronic configuration, molar mass, and polarity – see Table S1 for comparison). Therefore, differences in their adsorption mechanisms on graphene are expected, resulting in different resistance and spectral noise changes. Moreover, additional UV enhancement effects can be observed for graphene yielding highly different sensor responses compared to dark conditions.

**Figure 2:**
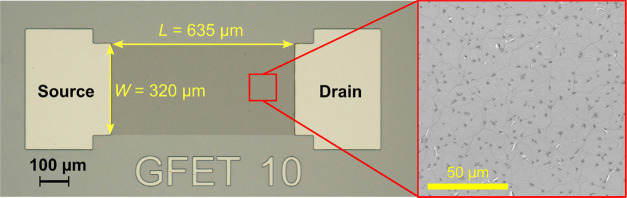
Confocal optical microscopy image of a GFET. The magnified image on the graphene channel reveals a significant number of point and edge defects.

We used graphene grown by the chemical vapor deposition (CVD) method on the surface of thin copper foils (www.graphenea.com) and transferred it from copper to SiO_2_/Si substrate using the procedure described in detail elsewhere.^21^Figure 2 shows the optical microscopy picture of the graphene back-gate FET (left panel) and a magnified image of the CVD-grown graphene layer. Optical characterization of the graphene sensing layer revealed a highly defective structure of Cu-grown graphene used in our experiments (after annealing in a vacuum). As shown in Figure 2, a significant number of cracks and point defects in the form of grains (with an average size of ∼1 μm) are visible in the structure. Nevertheless, previous Raman spectroscopy studies confirmed that CVD-graphene grown on Cu foil consists of a single layer.^22,23^ The observed morphological defects may be due to partial overlapping of graphene monolayers as well as cracks exposing their edges. The defective morphology results from graphene growth on Cu foil during the CVD process and further mechanical processing of graphene layer transfer on the SiO_2_/Si substrate. For detection, such defects create potential binding sites for ambient gas molecules.

### GFET Sensor DC Characteristics

**Figure 3:**
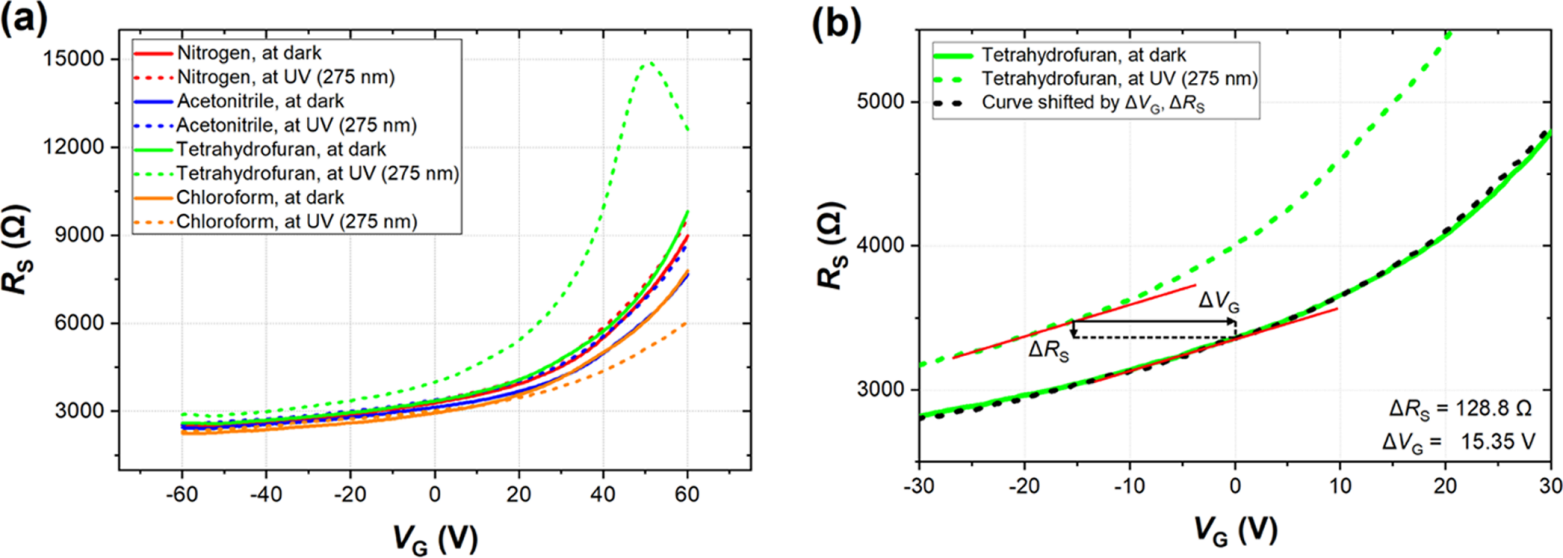
DC resistance *R*_S_ of the back-gated GFET sensor between the drain and source as a function of gate voltage *V*_G_ (a) for different gases in the dark and under UV irradiation (275 nm), and (b) a close-up for tetrahydrofuran, which shows how the UV light shifts both sensor resistance and gate voltage. The black dashed curve refers to the curve for tetrahydrofuran at UV (275 nm) after the shift by Δ*R*_S_ and Δ*V*_G_ marked by black arrows.

Before DC and noise measurements, the GFET sensor was cleaned at a high temperature (∼300 °C) in a vacuum (∼10^–7^ mbar) according to the description provided in Methods. The initial cleaning resulted in the increase of sensor resistance, *R*_S_, with a more pronounced change at higher positive gate voltages, with an ∼88% increase of *R*_S_ at *V*_G_ = 30 V. Characteristic drain–source current *I*_DS_ vs gate voltage *V*_G_ curves after GFET cleaning are depicted in Figure S1. Thermal annealing was earlier proved to increase the total graphene resistance and introduce defects serving as adsorption sites for gas sensing performance improvement.^24^ The *R*_S_ vs *V*_G_ characteristics for the different gases are presented in Figure 3. Additionally, Figure S2 shows the response of the GFET sensor presented as a relative change of sensor resistance in relation to the nitrogen case as a reference in the dark and under UV light for the broader presentation of collected data. As seen, in all cases, resistance increases with the increased gate voltage, which corresponds to the *p*-doped graphene. Nitrogen was used as a carrier gas in all measurements; thus, we consider the nitrogen case a reference in DC and noise studies. The solid red line in Figure 3a shows the *R*_S_ vs *V*_G_ characteristics of a GFET under nitrogen exposure. Measurements in acetonitrile, tetrahydrofuran, and chloroform were performed on three consecutive days after the cleaning procedure, which was required by the long recovery time of the graphene layer.

The sensor was kept in a desiccator under low vacuum overnight between subsequent days to limit its exposure to oxygen and humidity present in laboratory air and maintain the cleanliness of the sensor surface. Control measurement in laboratory air conditions was conducted each day to verify that the sensor recovers overnight (∼3% of resistance variance at *V*_G_ = 60 V during consecutive days).

The GFET sensor responds to each organic vapor differently. However, only for tetrahydrofuran do we see the distinct characteristic shift toward negative gate voltages. For acetonitrile, at *V*_G_ = 60 V, *R*_S_ decreases slightly, whereas it starts to increase under UV irradiation for the same gas. For chloroform, the sensor responds noticeably after UV irradiation by reducing the graphene resistance by ∼33% at *V*_G_ = 60 V and shifting the characteristic toward a more pronounced p-type conductance of the sensing layer. Interestingly, for tetrahydrofuran, the sensor response is the opposite. The gas increases *R*_S_ with a strong shift toward negative gate voltages under UV irradiation. A maximum *R*_S_ at *V*_G_ = ∼50 V is observed. The direction of changes indicates a strong n-type doping effect induced by tetrahydrofuran.

The differences between the sensor response to the organic gas molecules may be caused by the different chemical nature of the three organic compounds. Samnakay et al. reported that the polarity of organic vapors could explain differences in MoS_2_-based sensor responses.^25^ The opposite direction of current changes was observed for ethanol, methanol, and acetonitrile as polar solvents compared to chloroform and toluene – nonpolar compounds. In general, adsorption energies for organic molecules on graphene have been reported to be primarily governed by dispersion interactions (∼60%), even for polar molecules.^26^ For small molecules, including inorganic ones, adsorption energies on graphene were calculated to be relatively low.^27^ For aromatic molecules, calculations suggest that π–π stacking leads to about equal magnitude of dispersion and Coulomb interactions.^28^ Patil et al. showed that the geometric configuration of organic molecules significantly influences the adsorption energy.^29^ The authors demonstrated that molecules were physisorbed on graphene with negligible charge transfer for chloroform but that significant charge rearrangement occurred due to induced dipole interactions. Thus, the type of organic molecule, including functional groups, electronic configuration, polarity, *etc*., gives rise to different interactions between the adsorbate and graphene.

Chloroform is regarded as a nonpolar solvent because of its low polarity and small electrical permittivity, and yet it reduces the GFET sensor resistance, as observed in Figure 3a. The chlorine atoms in adsorbed chloroform have a partial negative charge,^29^ creating a depletion layer at the interface. Thus, hole accumulation in graphene may reduce resistance if the sensing material acts as a p-type semiconductor, as shown in Figure 3a. In the case of acetonitrile, which exhibits a weaker, mainly dispersive, interaction,^26^ the DC responses obtained in our study are relatively low. These sensor responses can be compared to calculated adsorption energies, which are reported to be in the range of 234 and 286 meV for acetonitrile^26^ and 357 meV for chloroform.^30^

To the best of the authors′ knowledge, no detailed quantification of adsorption of tetrahydrofuran on graphene is reported in the literature. However, the adsorption process with a ring-opening effect was reported for the solvent and conventional semiconductors.^31,32^ Tetrahydrofuran (THF) is a cyclic molecule with an oxygen atom in its structure (Table S1). Calculations show that it forms coordinated bonds and adsorbs either without or with ring-opening and form a bridge-like structure. The latter case is energetically favorable if the activation energy for ring-opening can be overcome.^32^ Charge transfer to the graphene substrate *via* the nucleophilic O atom could explain the significant increase of the GFET sensor resistance in Figure 3a. The direction of changes can be explained by electron injection into the graphene structure, which leaves the oxygen in tetrahydrofuran positively charged and increases the resistance of the p-type channel material. Figure S3 presents the GFET sensor recovery after tetrahydrofuran exposure. The reversible sensor characteristic suggests molecular THF bonding, *i.e*., no ring-opening reaction, upon adsorption under UV irradiation. The effect of a strong shift into the n-type channel is then attributed to the dative bonding of O in THF to graphene. This qualitative explanation requires, however, more in-depth studies. Some light is shed by the *in situ* FTIR spectra measurements discussed below.

The C=O bond in acetone can provide both an electrophilic C+ atom and a nucleophilic O– atom upon adsorption and lifting of the resonant double bond to a predominantly single-bonded species. As a result, depending on the adsorbate bonding configuration, C can act as an electrophile and O can act as a nucleophilic site. In contrast to THF, our measured DC characteristic shows a slight decrease in resistance upon acetone exposure, and no shift toward positive gate voltages was observed (Figure S4a). These results suggest that the electrophilic C atom in acetone dominates the sensor response, while for THF, the π-bonded C ring structure redistributes excess electrons, and charge transfer from the O atom in THF to graphene dominates the sensor response. Previous theoretical work shows that acetone adsorbs favorably with oxygen and hydrogen atoms above the hollow site of the carbon ring by weak van der Waals interactions and that acetone acts as an electron acceptor,^33,34^ qualitatively confirming the slightly decreased resistance seen in Figure S4a at high *V*_G_ voltages.

Returning to THF exposure of the GFET, a close-up in Figure 3b reveals another interesting observation. It has previously been reported that UV light can have a twofold effect on the DC characteristics, including the effect of photoconductivity and photogating.^35,36^ It is widely known that UV light can form electron–hole pairs that may participate in surface processes and gas detection on graphene. The photoconductive effect is related to the change of channel conductance due to charge carriers induced in graphene by UV light, resulting in a vertical shift of DC characteristics. On the other hand, the photogating, or photovoltaic, effect leads to photoinduced gate voltage, shifting the FET threshold voltage. Thus, the *R*_S_–*V*_G_ curves move left or right depending on the reducing or oxidizing properties of ambient gas. Assuming that these two effects are independent, we may decompose the DC characteristic, as shown in Figure 3b. Thus, a significant shift of *V*_G_ (15.35 V is about 50% of the considered *V*_G_ values) and only a slight change in *R*_S_ (128.8 Ω, resulting in a relative change of only ∼4%) are evident, suggesting that the dominating effect on the DC response upon UV irradiation of the THF-exposed GFET is the photogating effect. The same dominant photogating effect is also observed in the recovery phase (Figure S3).

FTIR measurements were performed to characterize THF adsorption on graphene (Figure S5). *In situ* transmission FTIR spectra reveal two absorption bands at ∼1000 and ∼850 cm^–1^ upon THF adsorption on graphene that can be attributed to the O–C–O stretching and out-of-plane C–H mode in THF, respectively. They are red-shifted compared to liquid THF (blue dotted line), signaling intramolecular C–O–C bond-weakening and charge redistribution of electrons in the ring structure upon adsorption, corroborating the DC sensor characteristics with electron transfer from the O atom in THF and concomitant charge redistribution in the ring structure.

### GFET Sensor Noise Characteristics

**Figure 4:**
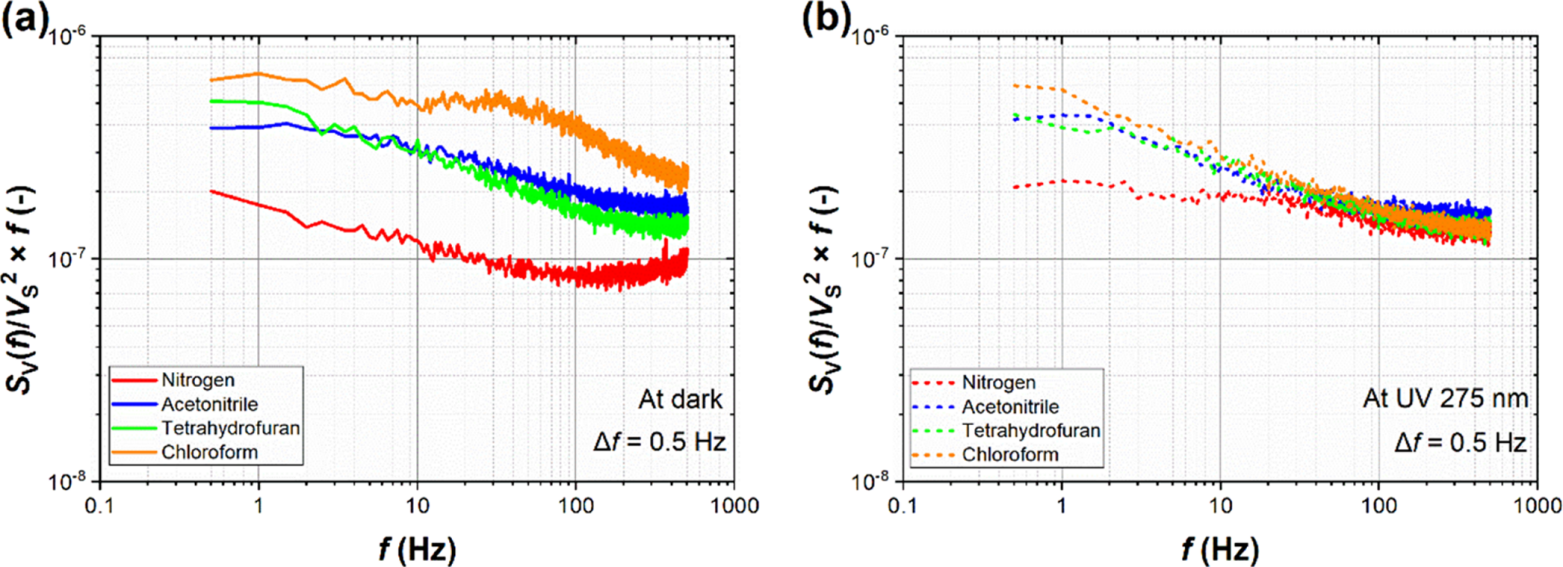
Power spectral density of voltage fluctuations *S*_V_(*f*) normalized to *V*_S_^2^ and multiplied by *f* for a GFET sensor, where *V*_S_ is the DC voltage and *f* is the frequency. Plots (a) and (b) show the spectral range between 0.5 Hz and 500 Hz for dark conditions and under UV irradiation, respectively.

Noise measurements were conducted to complement DC response studies. Figure 4 represents the normalized power spectral density of voltage fluctuations multiplied by the frequency in the dark and under UV irradiation, respectively, *viz*., *S*_V_(*f*)/*V*_S_^2^ × *f*. The noise spectra collected in the dark (Figure 4a) reveal characteristic bulges that differ for all three gases. For THF (green curve), a maximum occurs at the lowest frequencies of ∼1 Hz. Chloroform (orange curve) has the most distinct noise spectrum with maxima at ∼1 and ∼30 Hz. These characteristic frequencies change upon UV irradiation, leading to similarly shaped noise spectra for all three organic vapors, exhibiting differences only in the absolute noise level (Figure 4b). This shows that the UV light-induced changes of the power spectra dominate over gas adsorption and yield higher selectivity under dark conditions. Control noise measurements for acetone (Figure S4b) show a characteristic frequency maximum of the power spectrum at ∼1 Hz in the dark, like THF. However, since the DC responses are opposite for THF and acetone (increasing and decreasing resistance, respectively), we note that discrimination between these two gases is possible with combined DC and noise measurements, therefore enhancing the selectivity of the GFET sensor. Comparing our results with those reported by Balandin^14^ and Rumyantsev,^18^ we see that corner frequencies (frequencies at which a plateau occurs on the 1/*f* noise spectrum) occur in similar ranges with minor shifts, suggesting that the dominant factor is the active material itself, even though the active area in the case of our sensor (∼0.2 mm^2^) is at least a few thousand times larger than in abovementioned works. At the same time, larger sensing areas usually allow more accessible fabrication routes, making the process faster and cheaper.

### Aging and Refreshing of the GFET Sensor

**Figure 5:**
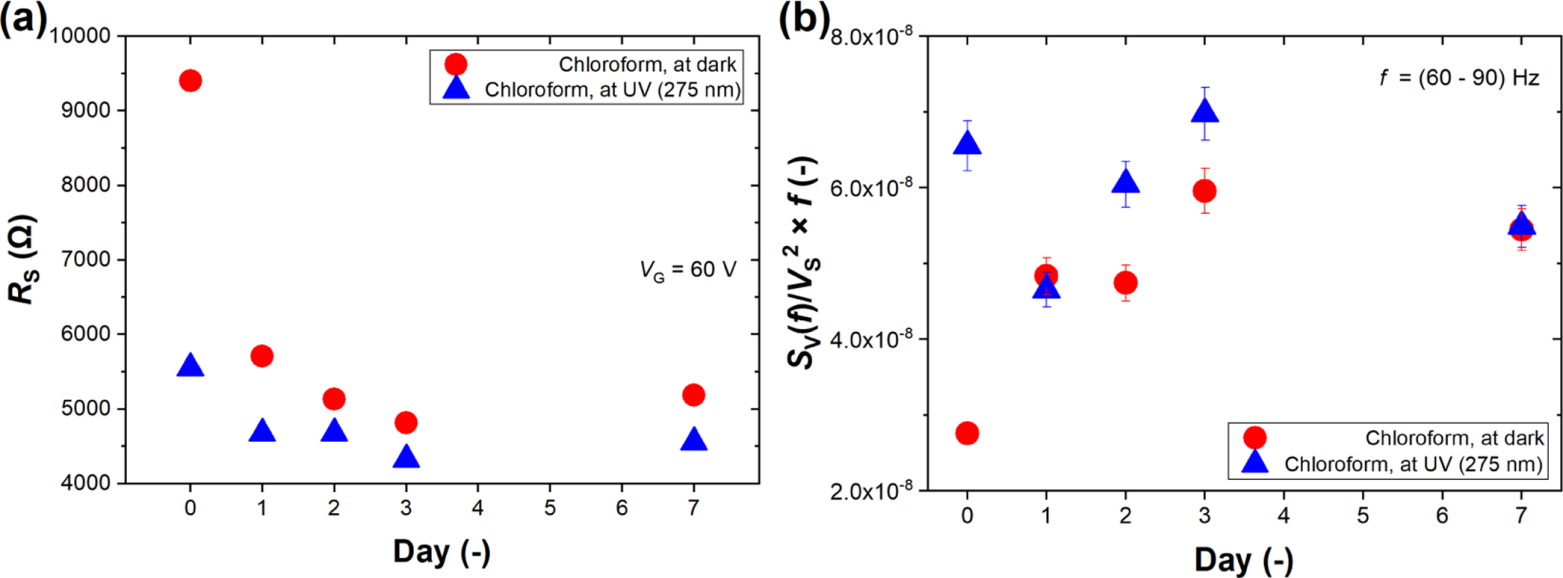
GFET sensor aging process after 7 days of exposure to chloroform vapor without intermittent cleaning of the sensor: (a) DC resistance *R*_S_ at gate voltage *V*_G_ = 60 V and (b) normalized power spectra *S*_V_(*f*)/*V*_S_^2^ × *f* of power spectral density of voltage fluctuations *S*_V_(*f*) for the GFET sensor with DC voltage *V*_S_ across its terminals as a mean value taken in the frequency *f* range of 60–90 Hz. The error bars in (b) present the standard deviation from the mean value. Results designated as 0 day refer to measurements conducted for the sensor just after the cleaning procedure described in the Methods section.

Next, we studied the GFET aging process by measuring *R*_S_ vs *V*_G_ characteristics and low-frequency noise spectra. Figure 5a depicts the sensor resistance at *V*_G_ = 60 V for seven consecutive days of chloroform gas exposure without additional sensor cleaning. Day 0 refers to a fresh, cleaned sensor that is highly responsive to chloroform, especially under UV light (baseline in nitrogen reached ∼10 kΩ on day 0). The sensor response does not recover to its pristine state during subsequent days. The baseline resistance at *V*_G_ = 60 V in nitrogen ranged between 5 and 6 kΩ for subsequent days. Much lower changes in *R*_S_ in the presence of chloroform under UV light are observed, whereas the sensor response remains almost intact in the presence of chloroform without UV irradiation. On the other hand, noise spectra collected during seven days were characterized by reproducible shapes with minor changes in noise level, as depicted in Figure 5b. The noise level increased during consecutive days in chloroform under dark conditions, whereas it oscillated around 6 × 10^–8^ when the sensor was exposed to both chloroform and UV irradiation. No strict dependence between the number of days and normalized power spectral density was observed. Thus, in contrast to DC measurements, the noise spectra exhibit a more repeatable sensor response for the back-gated GFET.

**Figure 6:**
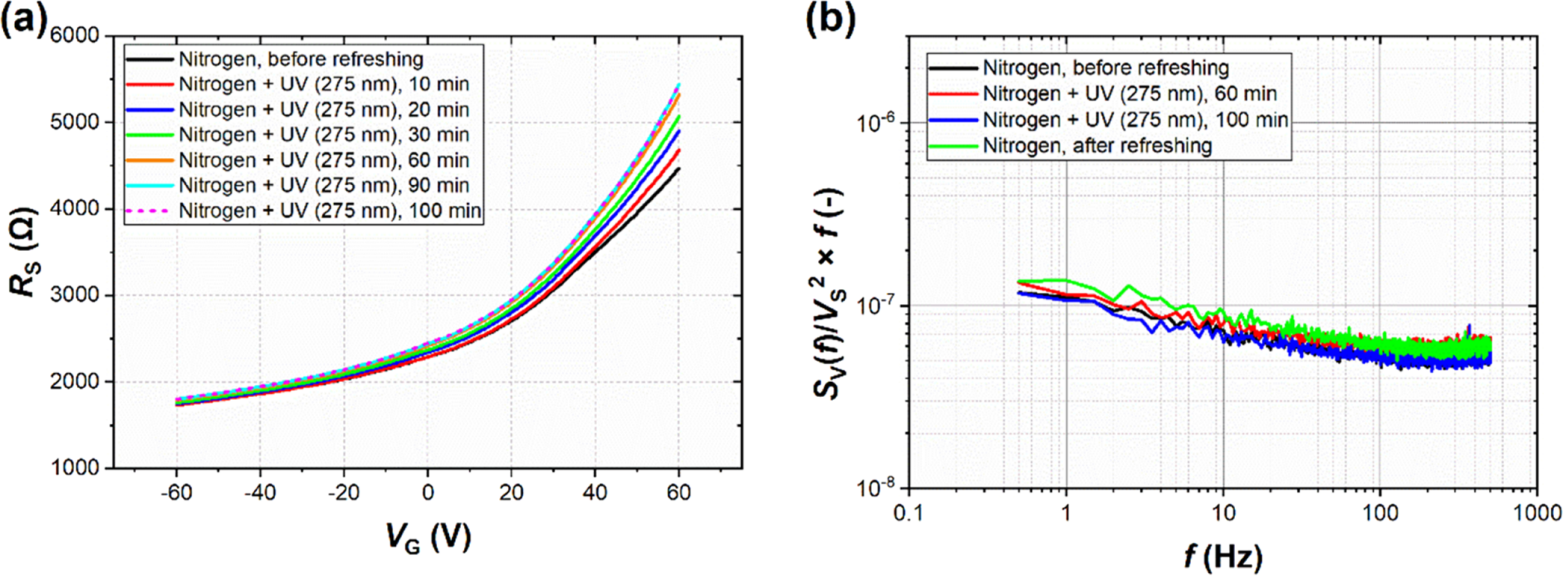
GFET refreshing process using UV irradiation under N_2_ ambient: (a) DC resistance *R*_S_ vs gate voltage *V*_G_ characteristics for different times of refreshing process and (b) normalized noise spectra product *S*_V_(*f*)/*V*_S_^2^ × *f* collected before, during, and after sensor refreshing for a total of 100 min.

Finally, we investigated the GFET sensor response after UV light exposure in an inert atmosphere of N_2_, which we refer to as a refreshing process. We employed UV irradiation with N_2_ purging after the repeated chloroform exposure measurements depicted in Figure 5. Figure 6a shows how *R*_S_ changes after a total of 100 min of refreshing. As expected, the fastest changes appear in the first 20 min. The curves after 90 and 100 min overlap each other, indicating that after such a long time, *R*_S_ stabilizes and attains a value close to that of a cleaned surface. UV irradiation removes previously adsorbed species (including oxygen or chloroform residues), and the inert atmosphere prevents readsorption of additional oxygen and humidity. Nevertheless, the cleaning effect with only UV light is not as efficient as heating under high-vacuum conditions. Figure 6b confirms that N_2_ and UV irradiation do not affect noise spectra, which supports our assumption that organic gases cause the characteristic features visible in the noise spectra in Figure 4.

## Conclusions

In this work, we studied CVD graphene back-gated FET sensor responses toward acetonitrile, tetrahydrofuran, chloroform, and acetone gases employing resistance vs gate voltage characteristics and 1/*f* noise measurements. The results presented in this work include GFET sensor performance in various ambient conditions, showing how selected technological (fabrication method, annealing, UV light enhancement) or aging processes influence the sensing performance. It is shown that the large effective sensing area of the investigated GFET of about 0.2 mm^2^ allowed analysis of characteristic features in the normalized noise spectra, which were different for all vapors and appeared in similar ranges as reported previously for graphene sensors employing thousand times smaller sensing areas. For acetonitrile and chloroform, the changes in the DC characteristics could be explained by the difference in their theoretical adsorption energies and their polarity. In the case of chloroform, the decrease in sensor resistance may be ascribed to its molecules binding favorably with negatively charged chlorine, creating a hole accumulation layer in *p*-doped graphene. Tetrahydrofuran significantly affected the sensor DC characteristic, shifting the *R*_S_ vs *V*_G_ curve by Δ*V*_G_ = 15.35 V and Δ*R*_S_ = 128.8 Ω. A dominant photogating effect suggests that a considerable number of charge carriers occur near the channel structure during the detection process. This creates a potential that acts as an additional gate voltage and shifts the characteristic curve toward negative *V*_G_ values. It is inferred that THF adsorbs nondissociatively (no ring-opening reaction, neither in the dark nor upon UV irradiation). A charge transfer reaction from the O atom in THF to graphene dominates the sensor response, leading to an increase in the resistance. Our studies also revealed that sensor aging mainly affects DC responses and not the noise spectra. We also confirm that annealing at high temperatures under low vacuum cleans the graphene surface more effectively than refreshing with only UV irradiation (275 nm) under a constant nitrogen flow.

The study shows that measurements of the GFET transfer characteristics combined with the FES method enhance the sensor sensitivity and selectivity. UV-assisted graphene-based sensors with reproducible features and low fabrication costs have the potential to develop a new group of highly sensitive, selective, and reversible gas sensors. By utilizing a well-defined combination of DC and noise studies, we propose a new direction in miniaturized gas detection systems by 2D materials. We believe there is still wide room for development in this area, but this path is undoubtedly worth noting and following in future scientific conquests.

## Methods

### Graphene Sensor Fabrication and Characterization

A low-resistivity silicon wafer was first cleaned and then put into a furnace chamber for ∼30 min at 1000 °C for thermal growth of SiO_2_. Afterward, an electrochemical delamination technique was employed to transfer PMMA-coated CVD graphene from copper foil (from Graphenea) onto the SiO_2_/Si substrate. This technique allows for the deposition of graphene on large areas. After the transfer process, the wafer was subsequently heated at 130 °C for 24 h and then rinsed in acetone to remove the PMMA layer. The graphene layer was patterned by laser lithography and etched by reactive-ion etching in oxygen plasma. Finally, source and drain contacts were patterned by laser lithography and fabricated by Ti/Au (5/150 nm thick) evaporation. Prior to back-gate contact deposition, SiO_2_ was removed from the backside of the Si wafer with HF. The backside of the wafer was metalized with Cr to serve as the back-gate of graphene-based field-effect transistors (GFETs). See Figure S6 for the schematic of the step-by-step GFET fabrication procedure. The channel length and width of the studied GFET were *L* = 635 μm and *W* = 320 μm, respectively. Optical characterization of the fabricated GFET was performed with a laser confocal microscope (Olympus, 3D LEXT OLS5100). *In situ* transmission FTIR spectra of graphene samples under gas and UV light exposure and liquid solvent as a reference were recorded with a Bruker 80v spectrometer.

### DC Resistance and Noise Measurements

A custom-prepared probe station with titanium needles was used to connect GFET electrodes with the measurement and bias units. A parameter analyzer (Keithley, type 4200A-SCS) with two source-measure units (Keithley, type 4201-SMU) was used for recording DC characteristics. Sensor resistance (*R*_S_) vs gate voltage (*V*_G_) characteristics were collected in the *V*_G_ range between −60 V and +60 V at selected operating conditions with 2 s hold time and the drain–source voltage bias set to 1 V. In the FES measurements, the sensor was connected in a circuit consisting of a low-noise operational amplifier and biased by a current source set to ∼90 μA (yielding a voltage across the sensor of ∼0.2 V). The gate voltage *V*_G_ was set to 0 V in the FES measurements. Such input parameters enabled measuring the 1/*f* noise originating from the material without interference from inherent noise from the measurement system. Figure S7 shows that the normalized power spectral density is nearly constant at the selected operating conditions, regardless of the input current. The power spectral density of voltage fluctuations caused by the input current flow was measured using a data acquisition board (National Instruments, type NI USB-4431) and a home-scripted LabVIEW program. During all measurements, the sensor was kept inside a metal shielding box to avoid external electromagnetic interference. For experiments with UV irradiation, a UV LED with maximum optical power at the wavelength of 275 nm was used (ProLight Opto, type PB2D-1CLA-TC) and positioned approximately 1 cm from the sensor surface, yielding an optical power density in the range between 1.06 and 1.59 mW cm^–2^.

### Gas Sensing Experiments

Annealing at high-vacuum conditions was employed to prepare GFET sensors for gas detection. The procedure included slow heating (at a rate of ∼2 °C min^–1^) and annealing the sensor at ∼300 °C for 30 min in a vacuum between about 1 × 10^–7^ and 5 × 10^–7^ mbar. After the heating process, the sensor was left in the vacuum chamber until cooling down to room temperature and transferred to the sensing chamber. In the gas sensor measurements, gas was admitted to the sample through a metal pipe connected to the gas distribution system, with the end pipe placed within 0.5 cm from the sample. Organic vapors were produced by feeding 50 mL/min of nitrogen (N_2_) gas through a glass beaker containing the selected organic liquid. Three organic solvents were used: acetonitrile, tetrahydrofuran, and chloroform. Additionally, acetone was used for supplementary studies. The constant overall gas flow was regulated by a mass flow controller (Analyst-MTC, series 358); therefore, the concentration of produced vapors admitted to the sample depended only on the N_2_ flow rate and vapor pressure of the organic liquid. The estimated concentrations varied for gases used in our experiments as follows: acetonitrile ∼156 ppm, tetrahydrofuran ∼100 ppm, chloroform ∼100 ppm, and acetone ∼110 ppm. For each cycle of measurements, the sensor was subjected to the selected gas for 20 min in dark conditions and then another 20 min with additional UV irradiation to obtain a steady state. All measurements were conducted at room temperature (RT ∼23 °C) and ambient pressure (∼1 bar).

## Supporting Information Available

Table with properties of selected organic vapors, current–voltage characteristic for the GFET in reference conditions, relative changes of sensor resistance vs gate voltage for selected organic gases, DC characteristics for the sensor recovery process after tetrahydrofuran introduction, sensing results for acetone (DC characteristics and noise spectra), FTIR spectra for graphene exposed to tetrahydrofuran, schematic of the GFET fabrication procedure, and dependence between the noise and sensor voltage at different input currents (PDF)
