# Supplementary material for: Organic Vapor Sensing Mechanisms by Large-Area Graphene Back-Gated Field-Effect Transistors under UV Irradiation
Source: ACS Sens. 2022 Sep 19;7(10):3094–101. doi: 10.1021/acssensors.2c01511 (PMC9623585; doi:10.1021/acssensors.2c01511)
Supplement: Supplementary file 1 — se2c01511_si_001.pdf [file se2c01511_si_001.pdf]

Supplementary Material

Organic Vapor Sensing Mechanisms by Large-Area Graphene Back-Gated Field-Effect Transistor under UV Irradiation

Katarzyna Drozdowska[a,*], Adil Rehman[b], Pavlo Sai[b], Bartłomiej Stonio[b,c], Aleksandra Krajewska[b], Maksym Dub[b], Jacek Kacperski[d], Grzegorz Cywiński[b], Maciej Haras[b,c], Sergey Rumyantsev[b], Lars Österlund[e], Janusz Smulko[a], Andrzej Kwiatkowski[a]

[a]*Department of Metrology and Optoelectronics, Faculty of Electronics, Telecommunications, and Informatics, Gdańsk University of Technology, G. Narutowicza 11/12, 80-233, Gdańsk, Poland*

[b]*CENTERA Laboratories, Institute of High Pressure Physics PAS, 01-142 Warsaw, Poland*

[c]*Centre for Advanced Materials and Technologies CEZAMAT, Warsaw University of Technology, 02-822 Warsaw Poland*

[d]*Institute of High Pressure Physics PAS, Sokołowska 29/37, 01-142, Warsaw, Poland*

[e]*Department of Materials Science and Engineering, The Ångström Laboratory, Uppsala University, P. O. Box, 75103 Uppsala, Sweden*

*Corresponding author – katarzyna.drozdowska@pg.edu.pl

Table S1 compares selected chemical properties and structures of the organic molecules used in the present GFET study. The distinct responses of the GFET sensor towards four vapors (especially tetrahydrofuran) suggest that the molecular structure and electronic bonding configuration influence the sensor response.

Table S1 Selected properties of acetonitrile, tetrahydrofuran, chloroform, and acetone.

**Compound**	**Chemical formula**	**Structural model**	**Relative polarity**[1,2]	**Relative electrical permittivity**[2,3]
Acetonitrile	C_2H_3N 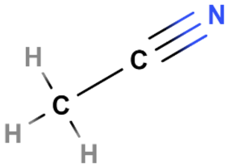	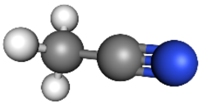	0.460	35.94
Tetrahydrofuran	C_4H_8O 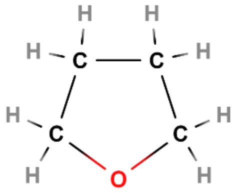	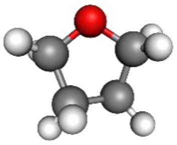	0.207	7.58
Chloroform	$CHCl_3$ 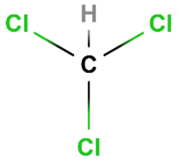	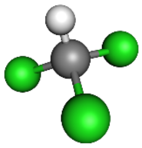	0.259	4.81
Acetone	C_3H_6O 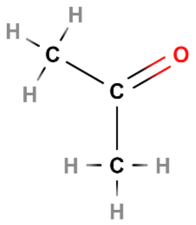	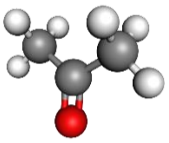	0.355	20.56

[1]Polarity in relation to water (relative polarity for water is by definition 1)

[2]Values extracted from Christian Reichardt, Solvents and Solvent Effects in Organic Chemistry, Wiley-VCH Publishers, 3rd ed., 2003

[3]Values for T = 25 °C

Figure S1 depicts characteristic drain-source current I_{DS} *vs* gate voltage V_G curves for GFET sensor in the presence of nitrogen (reference case in our studies). UV light slightly decreases the current flowing through the graphene channel in an inert atmosphere but does not shift the curve sideways noticeably.

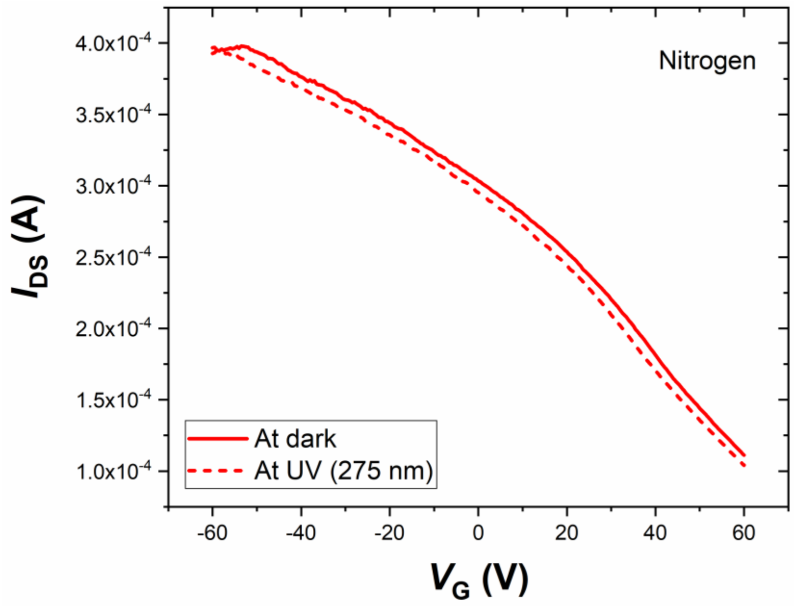

Figure S1. GFET drain-source current I_{DS} *vs* gate voltage V_G for reference conditions in nitrogen in the dark (solid line) and under UV (275 nm) irradiation (dashed line). The curves were measured after the cleaning procedure of graphene under a vacuum at 300 °C.

Figure S2 demonstrates GFET sensor response presented as a relative change of sensor resistance at selected ambiance in relation to the nitrogen atmosphere as follows:

$$\Delta R_S/R_0 = \frac{R_S - R_0}{R_0},$$

where R_S is the sensor resistance in the presence of selected gas, and R_0 is the sensor resistance in nitrogen only. In the dark, the absolute responses reach ~9% for tetrahydrofuran, ~13% for chloroform, and ~15 % for acetonitrile at V_G = 60 V. The sign of the responses reveals the direction of resistance changes, which is positive for tetrahydrofuran only. Under UV light, the major response with the local maximum is visible for tetrahydrofuran at ~50 V, which is ~25 times higher than for the dark conditions (0.04 in the dark and 1.02 under irradiation).

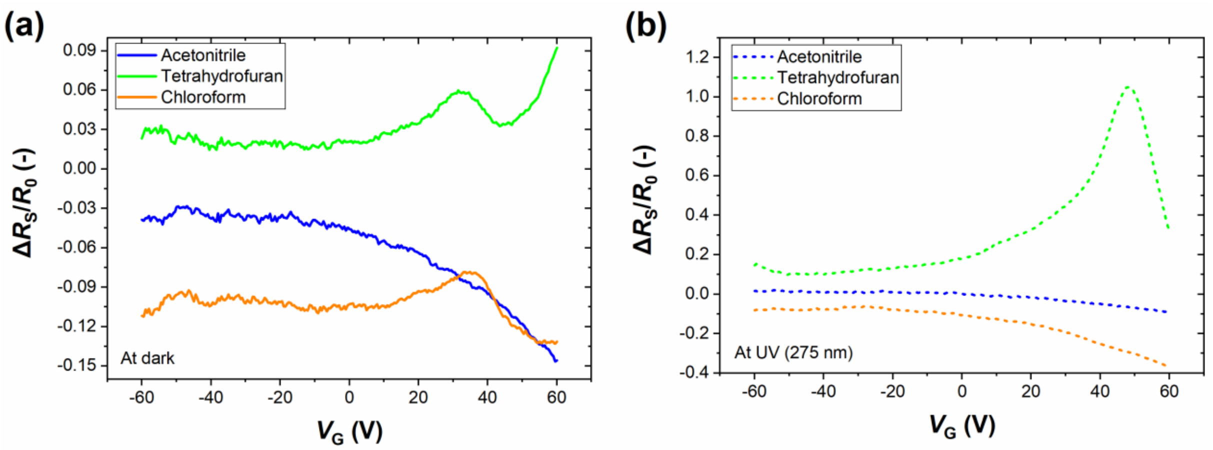

Figure S2. GFET sensor response in relation to nitrogen case as a reference **(a)** in the dark and **(b)** under UV (275 nm) irradiation.

Figure S3 presents the GFET sensor recovery process after tetrahydrofuran exposure. DC characteristics were collected every 5 min up to 1 h after deactivating UV irradiation and gas purging. The most significant changes occur in the first 15 min when the curve shifts towards negative gate voltages. One can see a pronounced shift of the maximum by ~3 V in the first 5 min of recovery. UV light promotes photoconductive and photogating effects, increasing the sensor resistance and shifting the curve to lower V_G (see Figure 3b). The shifts in Figure S3 suggest faster recovery from the photogating effect in the first minutes, followed by a slower recovery of the resistance (indicating recovery from the photoconductive effect). Overall, the results show that the GFET sensor requires a long time for full recovery since the baseline sensor resistance for the measurement shown in the figure was about 10 kΩ at V_G = 60 V in nitrogen, in the dark, before THF exposure and UV illumination. The samples were therefore kept overnight in a desiccator between consecutive detection cycles with different vapors to facilitate measurements.

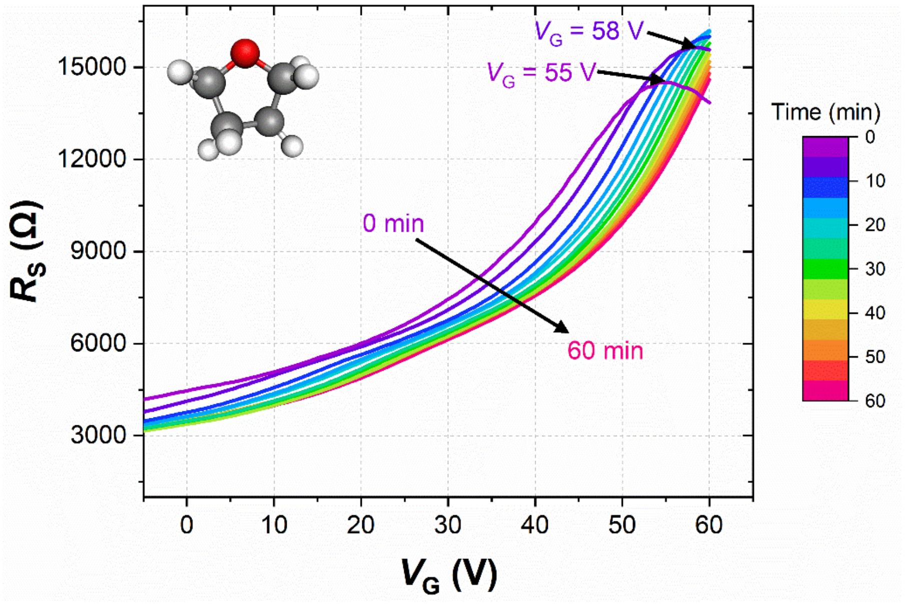

Figure S3. GFET recovery process presented as a DC resistance R_S of the back-gated GFET sensor between drain and source as a function of gate voltage V_G after tetrahydrofuran exposure. The characteristics were collected every 5 min for 60 min after switching off UV irradiation and THF gas exposure. The inset shows the THF molecule structure (O atom in red).

Figure S4 depicts a) DC and b) noise characteristics of the GFET sensor upon acetone exposure. Noise spectra reveal a peak with maximum visible at ~1 Hz for dark conditions only. The shape of the noise spectrum during UV irradiation (275 nm) remains similar to spectra recorded for acetonitrile, chloroform, and tetrahydrofuran under the same irradiation conditions.

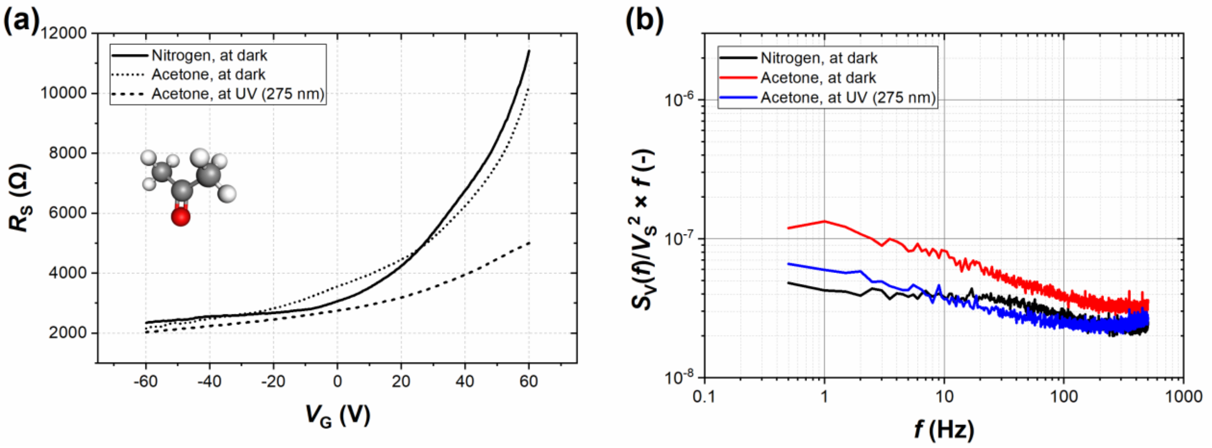

Figure S4. GFET sensor results for acetone: (a) DC resistance R_S of the back-gated GFET sensor between drain and source at selected gate voltages V_G, and (b) noise response, represented by the power spectral density of voltage fluctuations $S_V(f)/V_S^2$ normalized to the square of the sensor DC voltage V_S, and multiplied by the frequency f. The measurements were conducted in the dark and under UV light (275 nm). The inset in (a) illustrates the acetone molecular structure (O atom in red).

Figure S5 depicts *in situ* transmission FTIR spectra of graphene, THF/graphene, and liquid THF, respectively, recorded by a Bruker 80v spectrometer. The spectrum of graphene was recorded with the silicon substrate as the background (black dashed line). A graphene layer was transferred onto a thin undoped Si wafer and annealed at 300 °C in a high vacuum. The same graphene sample was subsequently exposed to N_2 and tetrahydrofuran (THF) at atmospheric pressure and UV irradiation (275 nm) for 20 min. The FTIR spectrum indicates that the graphene sample contains O atoms yielding carboxylate species at the surface. The spectrum after tetrahydrofuran (THF) adsorption and UV irradiation (solid red line) reveals two absorption bands at ~1000 cm^{-1} and ~850 cm^{-1} (marked by pink dashed vertical lines) that can be assigned to the O-C-O stretching and out-of-plane C-H mode in THF, respectively. The presented spectrum of liquid THF (blue dotted line) was recorded in a liquids holder with two ZnSe windows. We used the spectrum of empty liquids holder with two ZnSe windows as background in this case.

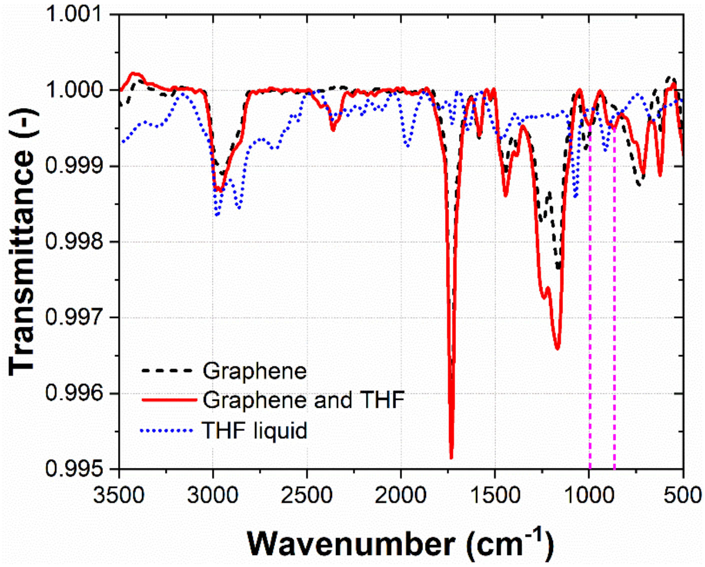

Figure S5. *In situ* transmission FTIR spectra recorded for graphene transferred onto a thin undoped silicon wafer pre-treated at 250 °C in a high vacuum (1.3x10^{-6} mbar) (dashed black line), the same graphene sample exposed to N_2 and tetrahydrofuran (THF) at atmospheric pressure and UV irradiation (275 nm) for 20 min. (solid red line), and liquid THF (dotted blue line).

Figure S6 depicts the schematic procedure for graphene-based FET (GFET) sensor fabrication. The procedure includes the following steps: delamination of Cu-grown graphene layer capped with PMMA, graphene transfer onto oxidized Si substrate, selective etching of graphene to produce a source-drain channel of specific dimensions, and metallization to produce source, drain, and back-gate chromium electrode.

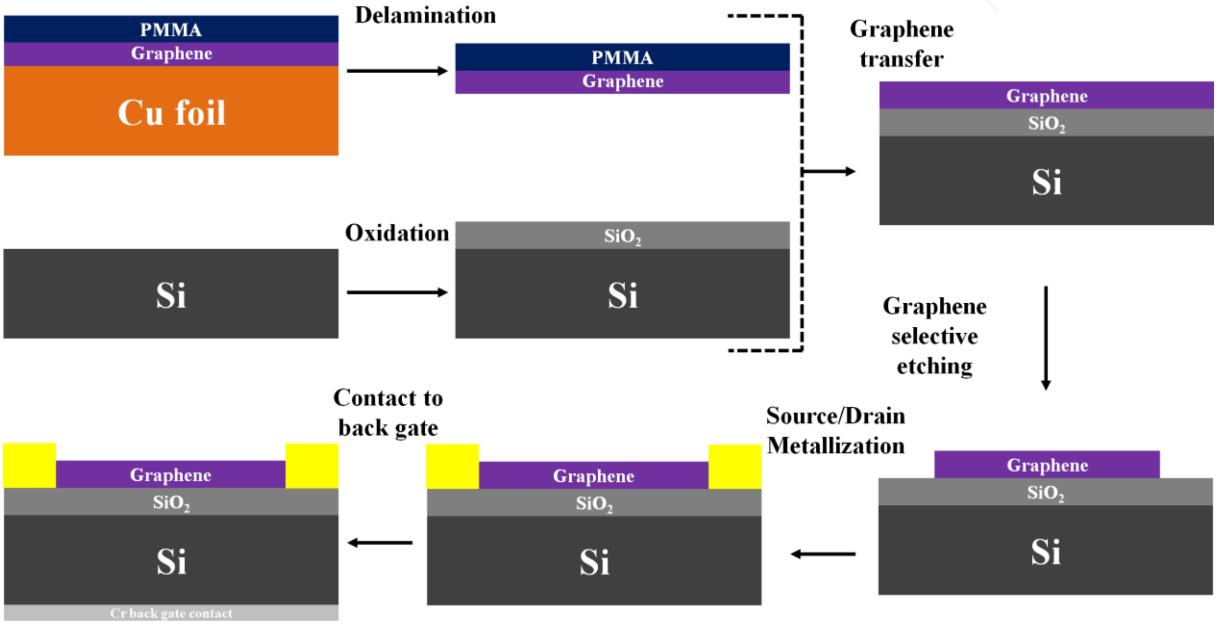

Figure S6. The procedure of graphene-FET (GFET) fabrication including several technological processes.

Figure S7 presents normalized power spectral density of voltage fluctuations $S_V(f_0)$ at frequency f_0 = 6 Hz as a function of sensor voltage squared V_S^2. The constant dependence between $S_V(f_0)/V_S^2$ and V_S^2 confirms that the measured noise signal is generated by fluctuation events in the investigated sensor. Therefore, 1/*f* noise observed for the measured sensor is not affected by the electronic components from the system (*e.g.*, contact needles, applied low-noise operational amplifier). The data points represent the noise measured for input currents from 15.3 µA to 96.4 µA, which induce a voltage across the sensor ranging from 0.038 V to 0.268 V.

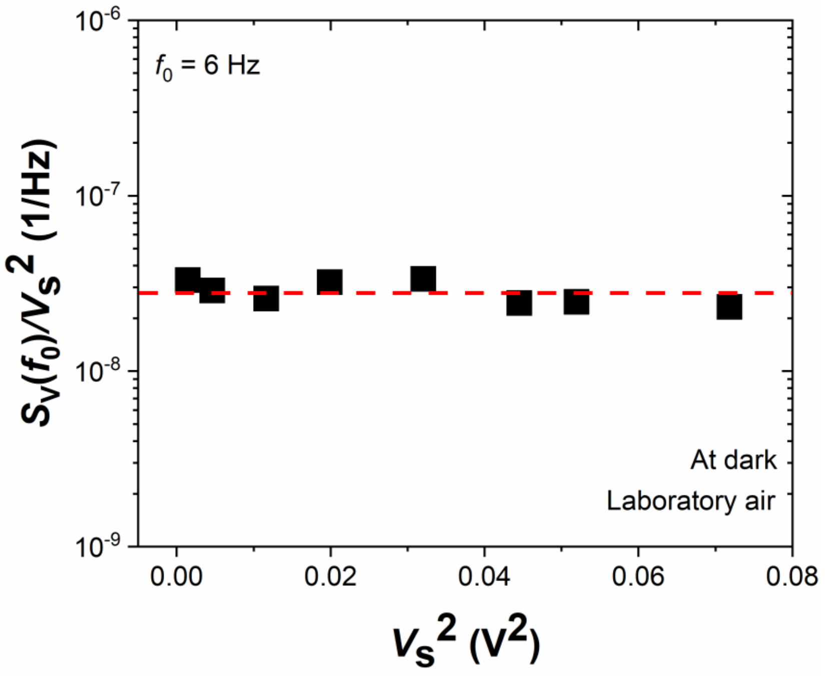

Figure S7. Power spectral density of voltage fluctuations $S_V(f_0)/V_S^2$ normalized to sensor voltage V_S at selected frequency f_0 = 6 Hz. The constant dependence between the noise product $S_V(f_0)/V_S^2$ and V_S^2 indicates that recorded noise is independent of measurement setup regardless of input current, and hence that 1/*f* noise is due solely to graphene.
